# Relative contributions of norspermidine synthesis and signaling pathways to the regulation of *Vibrio cholerae* biofilm formation

**DOI:** 10.1371/journal.pone.0186291

**Published:** 2017-10-18

**Authors:** Caitlin K. Wotanis, William P. Brennan, Anthony D. Angotti, Elizabeth A. Villa, Josiah P. Zayner, Alexandra N. Mozina, Alexandria C. Rutkovsky, Richard C. Sobe, Whitney G. Bond, Ece Karatan

**Affiliations:** Department of Biology, Appalachian State University, Boone, North Carolina, United States of America; Beijing Institute of Microbiology and Epidemiology, CHINA

## Abstract

The polyamine norspermidine is one of the major polyamines synthesized by Vibrionales and has also been found in various aquatic organisms. Norspermidine is among the environmental signals that positively regulate *Vibrio cholerae* biofilm formation. The NspS/MbaA signaling complex detects extracellular norspermidine and mediates the response to this polyamine. Norspermidine binding to the NspS periplasmic binding protein is thought to inhibit the phosphodiesterase activity of MbaA, increasing levels of the biofilm-promoting second messenger cyclic diguanylate monophosphate, thus enhancing biofilm formation. *V*. *cholerae* can also synthesize norspermidine using the enzyme NspC as well as import it from the environment. Deletion of the *nspC* gene was shown to reduce accumulation of bacteria in biofilms, leading to the conclusion that intracellular norspermidine is also a positive regulator of biofilm formation. Because *V*. *cholerae* uses norspermidine to synthesize the siderophore vibriobactin it is possible that intracellular norspermidine is required to obtain sufficient amounts of iron, which is also necessary for robust biofilm formation. The objective of this study was to assess the relative contributions of intracellular and extracellular norspermidine to the regulation of biofilm formation in *V*. *cholerae*. We show the biofilm defect of norspermidine synthesis mutants does not result from an inability to produce vibriobactin as vibriobactin synthesis mutants do not have diminished biofilm forming abilities. Furthermore, our work shows that extracellular, but not intracellular norspermidine, is mainly responsible for promoting biofilm formation. We establish that the NspS/MbaA signaling complex is the dominant mediator of biofilm formation in response to extracellular norspermidine, rather than norspermidine synthesized by NspC or imported into the cell.

## Introduction

Biofilms are communities of microbes that exist attached to surfaces and/or each other. In most cases, biofilm microbes are encased in a self-produced hydrated matrix of an extracellular polymeric substance (EPS) [1]. The major components of the EPS are polysaccharides, proteins, and DNA [2]. Bacteria may form biofilms as a survival mechanism as such structures have been shown to aid in protection against pH extremes, osmotic stress, UV radiation, antimicrobials, and the host’s immune response [3–6]. Biofilm formation is regulated by a variety of cues which include nutrient availability, quorum sensing, surface composition, osmolarity, and small molecules such as polyamines [7,8].

Polyamines are flexible aliphatic chains containing two or more amine groups that are positively charged at physiological pH. These natural polycations are involved in cell growth and development of both prokaryotic and eukaryotic cells [9]. While polyamines are believed to perform different functions in different organisms, they have been shown to play a role in biofilm formation in a variety of bacteria, including *Vibrio cholerae*, the causative agent of the diarrheal disease cholera [8,10]. Norspermidine is a relatively uncommon polyamine that is produced mainly by the Vibrionales [11,12]. It is also found in some extremophilic Archaea, in some aquatic and terrestrial plants, and lower aquatic eukaryotes including some diatoms, algae, arthropods, mollusks, and sea squirts [13–17].

**Fig 1:**
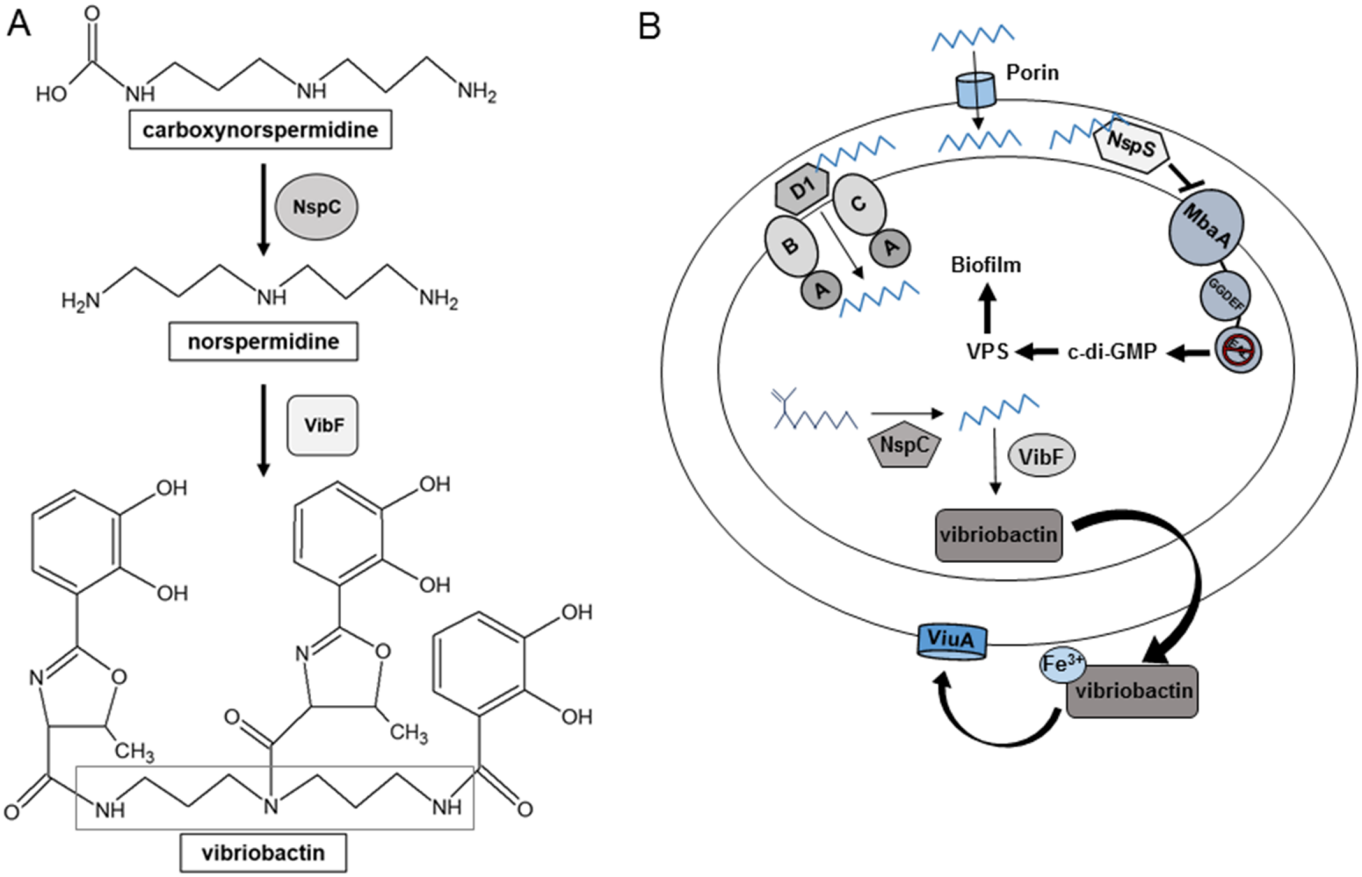
Norspermidine related processes in *V*. *cholerae*. **(A)** Synthesis of norspermidine and vibriobactin. Norspermidine can be synthesized by the enzyme NspC through decarboxylation of carboxynorspermidine. Norspermidine is utilized by VibF to form the backbone of the siderophore vibriobactin. Norspermidine backbone outlined by the grey box.**(B)** Norspermidine synthesis, utilization, transport, and signaling pathways. Norspermidine can be synthesized by NspC and can also be imported from the environment presumably through the PotABCD1 ABC-type transporter. Vibriobactin, synthesized from norspermidine, is secreted into the environment and binds to iron. This ferric-vibriobactin complex is recognized by the outer membrane protein ViuA and transported into the periplasm, which is then imported into the cell by an ABC-type transporter (not shown). Exogenous norspermidine is sensed by the NspS/MbaA signaling complex, which leads to increased VPS production and biofilm formation, presumably through increasing c-di-GMP levels in the cell. Norspermidine is represented by a zig-zag and carboxynorspermidine is represented by a branched zig-zag. VPS, *Vibrio* polysaccharide. The PotABCD1 ABC-type transporter is denoted as A, B, C, and D1.

Norspermidine is synthesized in *V*. *cholerae* through the decarboxylation of carboxynorspermidine by the enzyme NspC, encoded by the *nspC* gene (Fig 1A). Deletion of *nspC* was shown to reduce biofilm formation, which could be rescued by uptake of norspermidine from the growth medium [10]. Norspermidine import into the cell is mediated by the ABC transporter binding protein, PotD1, presumably together with the PotA, PotB, and PotC proteins, which comprise the rest of the transporter (Fig 1B) [18]. It has been suggested that adequate levels of cytoplasmic norspermidine are required for supporting robust biofilm formation by an unknown mechanism in *V*. *cholerae* [10]. Norspermidine forms the backbone of the *V*. *cholerae* siderophore vibriobactin and iron deficiency has been shown to inhibit biofilm formation [19,20]. Therefore, one possible mechanism by which intracellular norspermidine affects biofilm formation may involve iron acquisition; however, the effect of vibriobactin synthesis on biofilm formation has not been studied.

Norspermidine also acts as an extracellular signal that enhances *V*. *cholerae* biofilm formation [21]. The effect of norspermidine is mediated by the periplasmic binding protein NspS, which has been shown to bind norspermidine *in vitro* [18]. NspS itself is also an activator of biofilm formation. NspS is believed to interact with the GGDEF-EAL family protein MbaA, a cyclic diguanylate monophosphate (c-di-GMP) phosphodiesterase, and down-regulate its enzymatic activity, leading to increased c-di-GMP levels. Increased c-di-GMP activates expression of the *vps* (*V**ibrio* polysaccharide) genes encoding proteins responsible for the production of the biofilm polysaccharide in *V*. *cholerae*, which in turn increases biofilm formation. Norspermidine binding to NspS is believed to enhance the inhibitory effect of NspS on MbaA, leading to further increases in biofilm formation [8,18]; (Fig 1B).

The purpose of the current study was to delineate the interplay of norspermidine synthesis, transport, utilization, and signaling pathways on the regulation of biofilm formation in *V*. *cholerae*. We constructed various mutants in norspermidine-related pathways and assessed the resulting biofilm phenotype as well as cellular polyamine content in *V*. *cholerae*. We demonstrate that the NspS/MbaA signaling pathway is epistatic over norspermidine synthesis, import, and utilization pathways in regulating biofilms.

## Materials and methods

### Bacterial strains, plasmids, and media

The *V*. *cholerae* strain used was O139 MO10. More information on the bacterial strains and plasmids used in this study can be found in Table 1. Primers are listed in Table 2. All experiments were done in Luria-Bertani (LB) broth. To generate iron-deplete conditions, the ferric iron chelator ethylenediamine di(*ortho*-hydroxyphenylacetic acid) (EDDA) was added to LB at a concentration of 100 μg/ml after being deferrated according to the method of Rogers [22]. Streptomycin, ampicillin, and kanamycin were used at 100 μg/ml unless otherwise specified; tetracycline was used at 2.5 μg/ml. Primer synthesis and DNA sequencing were performed by Eurofins MWG Operon.

**Table 1:** Bacterial strains and plasmids.

Strain	Genotype	Reference/source
***E*. *coli***		
DH5α	F– Φ80*lac*ZΔM15 Δ(*lac*ZYA-*arg*F) U169 *rec*A1 *end*A1 *hsd*R17 (rK–, mK+) *pho*A *sup*E44 λ– *thi*-1 *gyr*A96 *rel*A1	Invitrogen
DH5αλpir	*sup*E44, Δ*lac*U169 *hsd*R17, *rec*A1 *end*A1 *gyr*A96 thi-1 relA1, λpir	[23]
SM10λpir	*thi thr leu tonA lacY supE recA*::*RP4-2-Tc*::*MuλpirR6K;Km*^*R*^	[24]
***V*. *cholerae***		
PW249	MO10, clinical isolate of *V*. *cholerae* O139 from India, Sm^R^	[25]
PW357	MO10 *lacZ*::*vpsLp* → *lacZ*, Sm^R^	[26]
PW514	PW357Δ*nspS*, Sm^R^	[8]
PW444	PW357Δ*mbaA*, Sm^R^	[8]
AK007	PW514 with pACYC184, Tet^R^, Sm^R^	[33]
AK149	PW357Δ*nspS*, *ΔpotD1*, Sm^R^	[18]
AK160	PW357Δ*potD1*, Sm^R^	[21]
AK164	AK160 with pACYC184, Tet^R^, Sm^R^	This study
AK165	AK160 with pAR2, Tet^R^, Sm^R^	This study
AK192	PW514 with pNP1, Tet^R^, Sm^R^	[33]
AK297	PW357 with pACYC184, Tet^R^, Sm^R^	This study
AK314	PW357*nspC*::*kan*^*R*^, Kan^R^, Sm^R^	[18]
AK317	PW357*nspC*::*kan*^*R*^, Δ*potD1*, Kan^R^, Sm^R^	[18]
AK335	AK314 with pACYC184, Tet^R^, Sm^R^, Kan^R^	This study
AK361	PW357Δ*vibF*, Sm^R^	This study
AK366	PW357*viuA*::*tet*^*R*^, Tet^R^, Sm^R^	This study
AK400	PW357*nspC*::*kan*^*R*^, Δ*vibF*, Kan^R^, Sm^R^	This study
AK470	PW357*nspC*::*kan*^*R*^, Δ*nspS*, Kan^R^, Sm^R^	This study
AK487	PW357*nspC*::*kan*^*R*^, Δ*nspS*, Δ*potD1*, Kan^R^, Sm^R^	This study
AK672	PW357*nspC*::*kan*^*R*^, Δ*mbaA*, Kan^R^, Sm^R^	This study
AK689	PW357 with pEVS143, Kan^R^, Sm^R^	This study
AK779	PW357Δ*nspS*Δ*vibF*, Sm^R^	This study
AK692	PW514 with pEVS143, Kan^R^, Sm^R^	This study
AK695	PW514 with pCMW75, Kan^R^, Sm^R^	This study
AK698	PW514 with pCMW98, Kan^R^, Sm^R^	This study
AK703	AK470 with pNP1, Tet^R^, Sm^R^	This study
AK714	AK487 with pEVS143, Kan^R^, Sm^R^	This study
AK717	AK487 with pCMW75, Kan^R^, Sm^R^	This study
AK720	AK487 with pCMW98, Kan^R^, Sm^R^	This study
AK739	AK672 with pEVS143, Kan^R^, Sm^R^	This study
AK741	AK672 with pVC0703, Kan^R^, Sm^R^	This study
AK743	AK470 with pMM13, Tet^R^, Sm^R^	This study
AK804	AK314 with pMM13, Tet^R^, Sm^R^, Kan^R^	This study
**Plasmid**		
pCR2.1-TOPO	Plasmid for TOPO cloning, Ap^R^	Invitrogen
pWM91	*oriR*6k, *lacAα*, *sacB*, homologous recombination plasmid, Ap^R^	[27]
pACYC184	Cloning plasmid, low copy, Tet^R^, Cm^R^	New England Biolabs
pCMW75	pEVS143::*qrgB* (*Vibrio harveyi* DGC), overexpression vector, Kan^R^	[28]
pCMW98	Active-site mutant of *qrgB* in pCMW75	[28]
pEVS143	Broad-host-range cloning vector; inducible Cm^R^ and GFP; Kan^R^	[29]
pVC0703	pEVS143::*mbaA*	[30]
pWCW3	pCVD442 with SalI-SacI fragment from pWCW2 containing 882-bp in-frame deletion of *vibF*, Ap^R^	[31]
pPAC20	pCVD442 with 4.2-kbp PvuII fragment containing *viuA*::*tet*^*R*^, Ap^R^, Tet^R^	[32]
pAR17	pWM91 carrying an internal 981 bp fragment of *nspC* replaced with kanamycin acetyltransferase gene	[18]
pMM9	pWM91 containing an internal in-frame deletion of *potD1*	[21]
pNP1	pACYC184::*nspS*	[33]
pMM13	pACYC184::*nspC*	[34]
pAR2	pACYC184::*potD1*	This study

**Table 2:** Primers.

Primer	Description	Sequence (5’-3’)
PA138	Forward primer for cloning *potD1*	5’- ACGCCTAGTTAGGTTCTTTC-3’
PA144	Reverse primer for cloning *potD1*, encodes a V5 tag	5’CCATGGCTACGTAGAATCGAGACCGAGGA AGGGTTAGGGATAGGCTTACCGCCGCTGC CGCTGCCATCGTTCACTTTTAGCTTTTGG-3’
PA209	Forward primer for *vibF* internal deletion confirmation	5’-GTGTTGGCTGCGTTCGTGAC -3’
PA210	Reverse primer for *vibF* internal deletion confirmation	5’-GGGGTCAGTGGCATCTCCTG-3’
PA223	Forward primer for *viuA*::*tet*^*R*^ confirmation	5’- CGCAAACAGCGGGTATGATC-3’
PA224	Reverse primer for *viuA*::*tet*^*R*^ confirmation	5’- AAGGCTAGTCCTGCCCCACTC-3’

### Construction of mutants

Mutants were constructed by double homologous recombination with sucrose selection as described by Metcalf *et al*. [27]. The *viuA*::*tet*^*R*^ mutant was constructed by mating *E*. *coli* SM10λpir containing pPAC20 with wild-type *V*. *cholerae* to generate the *V*. *cholerae viuA*::*tet*^*R*^ mutant. *E*. *coli* SM10λpir containing pWCW3 was mated with wild-type *V*. *cholerae* or with the *V*. *cholerae* Δ*nspS* mutant to generate the *V*. *cholerae* Δ*vibF* and Δ*nspS*Δ*vibF* strains, respectively. *E*. *coli* SM10λpir containing pAR17 [18] was mated with the *V*. *cholerae* Δ*vibF* mutant to generate the *V*. *cholerae nspC*::*kan*^*R*^*ΔvibF* mutant. *E*. *coli* SM10λpir containing pAR17 [18] was mated with the *V*. *cholerae ΔnspS* mutant or the *V*. *cholerae* Δ*mbaA* mutant to generate *V*. *cholerae nspC*::*kan*^*R*^*ΔnspS* and *nspC*::*kan*^*R*^Δ*mbaA* mutants, respectively. *E*. *coli* SM10λpir containing pMM9 [21] was mated with the *V*. *cholerae nspC*::*kan*^*R*^Δ*nspS* mutant to generate *nspC*::*kan*^*R*^Δ*nspS*Δ*potD1*. After mating, strains were streaked on selection plates containing 100 μg/ml streptomycin and 50 μg/ml ampicillin. For generation of the *viuA*::*tet*^*R*^ and *nspC*::*kan*^*R*^ strains, selection plates also contained tetracycline (2.5μg/ml) or kanamycin (50 μg/ml). Single colonies were restreaked on selection plates to confirm single cross-over events followed by streaking on LB plates without antibiotics in order to facilitate a second recombination event. After incubation overnight, multiple isolated colonies were streaked on sucrose plates without antibiotics or containing tetracycline (2.5 μg/ml) or kanamycin (50 μg/ml) if relevant and incubated for 48 hours. Isolated colonies were patched on LB plates containing either streptomycin (100 μg/ml) or ampicillin (50 μg/ml) in order to screen for streptomycin resistance and ampicillin sensitivity and confirming successful homologous recombination. Colonies that grew on streptomycin but not ampicillin plates were confirmed for the recombination event by colony PCR. Primers used to verify construction of the *ΔvibF* and *viuA*::*tet*^*R*^ mutants are listed in Table 2. Primers used to verify Δ*potD1 and nspC*::*kan*^*R*^ have been published in [21] and [18], respectively.

### Cloning of the *potD1* gene

Primers PA138 and PA144 were used to amplify the *potD1* gene from *V*. *cholerae* chromosomal DNA. This PCR product was first TOPO-cloned into the pCR2.1 vector (Invitrogen) and correct sequence was verified. It was then excised with EcoRI and NcoI and subcloned into pACYC184 digested with the same restriction enzymes generating pAR2.

### Extraction, benzoylation, and detection of polyamines

Bacteria were grown at 27°C to mid-exponential phase, pelleted, washed twice with 1X PBS, and resuspended in 10 μl water per milligram wet cell weight. Then, 250 μl of the cell suspension corresponding to 25 mg of cells was lysed using sonication and the cell debris was removed by centrifugation. Cellular proteins were precipitated with 50% (w/v) trichloroacetic acid and centrifuged. The supernatant containing the polyamines was removed and benzoylated as described previously [21]. For measurement of polyamines in the spent medium, cultures were centrifuged, 1 ml of the media was removed and passed through a 0.22 μm syringe filter to remove any remaining cells, proteins were precipitated with trichloroacetic acid, and the supernatant was for polyamine extraction. Briefly, samples were extracted twice with chloroform, evaporated to dryness, and dissolved in 100 μl of 60% methanol in water. A standard mix containing 0.1 mM of each polyamine was also prepared and benzoylated each time. The set of benzoylated polyamine samples were separated using a Phenomenex Spherclone ODS column (5 μm, 250 x 4.6 mm) that was fitted with a 4.0 x 3.0 mm guard cartridge with the system described above. The runs were performed using a gradient of 45–60% methanol in water for 30 minutes with a 10-minute isocratic equilibration of 45% methanol in water.

### Biofilm assays

Bacteria were diluted in 0.3 ml LB at an OD_655_ of 0.02 taken using a Bio-Rad iMark MicroPlate Reader and incubated in borosilicate test tubes for either 18, 24, or 34 hours at 27°C or 37°C without shaking. After 18, 24, or 34 hours, planktonic cells were discarded, and the biofilm was washed once with 0.3 ml of 1x PBS. The biofilm was then mechanically disrupted in 0.3 ml of 1x PBS by vortexing with glass beads, and the cell density was measured at OD_655_ or OD_595_ depending on the availability of the filter. In assays using the pEVS143 vector system, we found that there was a lot of day to day variation in biofilm formation; therefore, the output was reported as percent wildtype to illustrate the trends in the data better. Three independent colonies were used for each experiment. All experiments were performed in triplicate and repeated multiple times to ensure reproducibility. Student’s t-tests were used to detect significant differences between various strains and treatments.

### Source data

All the data used to generate the figures and supplemental figures in this study can be found in Supplemental file (S1 File).

## Results

### Biofilm deficiency of *nspC* mutants is not due to a defect in vibriobactin production

**Fig 2:**
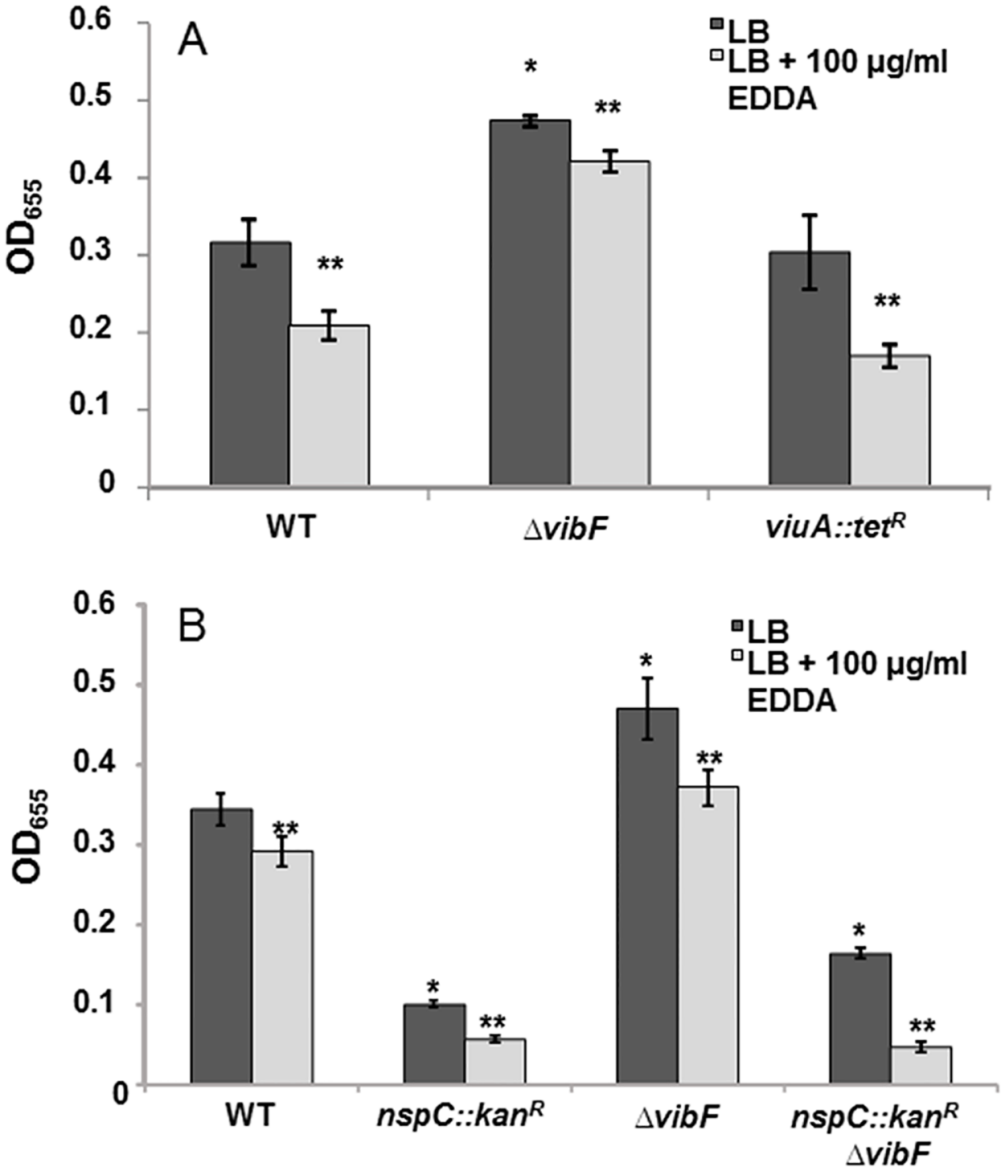
Effects of vibriobactin synthesis and utilization on biofilm formation in *V*. *cholerae*. **(A)** Biofilm formation of *ΔvibF* and *viuA*::*tet*^*R*^ mutants. **(B)** Biofilm formation of *ΔvibF*, *nspC*::*kan*^*R*^, and *nspC*::*kan*^*R*^*ΔvibF* mutants. Biofilms were formed in borosilicate tubes in LB broth for 24 h at 27°C and quantified as described in Materials and Methods. EDDA was added to chelate iron to generate iron-deplete conditions. Error bars show standard deviations of three biological replicates. A star indicates a statistically significant difference between wild type and the mutants. A double star indicates a statistically significant difference between growth media conditions. A p-value <0.05 was considered significant. WT, wild type.

In *V*. *cholerae*, norspermidine forms the backbone of the siderophore vibriobactin. Vibriobactin chelates ferric iron and ultimately transports the iron into the cell [20,35]. Vibriobactin is synthesized by three proteins: VibF, VibB, and VibH. VibB and VibH act as courier proteins by bringing the vibriobactin precursors to VibF, which is responsible for attaching oxazoline rings to a norspermidine backbone (Fig 1B); [20]. Like most other bacteria, *V*. *cholerae* requires iron for various cellular processes that are necessary for cell growth and survival [36]. In addition, low iron levels inhibit biofilm formation [19]. The enzyme carboxynorspermidine decarboxylase, which catalyzes the last step of norspermidine synthesis in *V*. *cholerae* is encoded by the *nspC* gene [10]. Deletion of *nspC* has been shown to lead to a reduction in biofilm formation; however, the mechanism behind this effect has not been elucidated [10]. We hypothesized that the decreased ability of the *nspC* mutant to form biofilms may be due to the inability of these strains to synthesize vibriobactin, resulting in reduced iron levels in the cell. To test this hypothesis, we first constructed two mutants defective in vibriobactin-mediated iron uptake into cells, Δ*vibF* and *viuA*::*tet*^*R*^. The Δ*vibF* mutant is not capable of synthesizing vibriobactin whereas the *viuA*::*tet*^*R*^ mutant has a disruption in the gene encoding the outer membrane receptor required for uptake of the vibriobactin-Fe^3+^ complex into the periplasm. As seen previously with the wild type, iron depletion resulted in a decrease in accumulation in biofilms for all of the strains [19]. However, neither of the mutants showed a reduction in biofilm levels in iron-replete or iron-deplete conditions as compared to the wild type (Fig 2A). To our surprise, deletion of *vibF* generated a 56 and 64% increase in biofilm formation in iron-deplete and iron-replete conditions, respectively, whereas biofilm forming ability of the *viuA* mutant was similar to the wild-type strain in both conditions (Fig 2A). These results indicate that the reduction in biofilms in *nspC* mutants is not a result of defective vibriobactin production. Furthermore, inability to synthesize but not to import vibriobactin enhances biofilms.

In a *vibF* mutant, norspermidine is not converted to vibriobactin and may therefore accumulate in the cell. However, in a *viuA* mutant norspermidine is utilized to synthesize vibriobactin, which is then transported out of the cell. We hypothesized the increase in biofilm formation of the Δ*vibF* mutant could be due to the accumulation of norspermidine in the cell in the absence of the VibF protein. To determine whether this increase required the synthesis of norspermidine, we constructed a mutant defective in both norspermidine and vibriobactin synthesis, *nspC*::*kan*^*R*^Δ*vibF*, and assessed its biofilm forming ability. Both the *nspC*::*kan*^*R*^Δ*vibF* and *nspC*::*kan*^*R*^ mutants exhibited a 40% decrease in biofilm formation in iron-replete conditions compared to wild type. Biofilm formation was reduced even further in iron-deplete conditions (Fig 2B). To further confirm that the altered biofilms seen in the *nspC*::*kan*^*R*^ and Δ*vibF* mutants are due to the deletions in these genes rather than unintended alterations elsewhere in the genome, we attempted to complement these mutants by introducing the genes on a plasmid. The *nspC*::*kan*^*R*^ mutant containing the empty vector was reduced in its ability to form biofilms compared to the wild-type bacteria (S1 Fig). While this difference was not statistically significant, the trend was the same in each biological replicate. Reintroducing *nspC* from a plasmid led to a very large increase in biofilm formation, which was higher than that formed by the wild type. We have previously reported that introducing this plasmid into wild-type cells also has the same effect on biofilm formation; the reason for this is not known [34]. Nevertheless, these experiments show that reintroducing the *nspC* gene corrects the inhibition of biofilm formation. We were not successful in cloning the *vibF* gene despite multiple attempts; hence, we were not able to perform complementation experiments. Therefore, while it is highly unlikely that the altered biofilm phenotype is a result of an unintended alteration in the genome, this still remains a possibility.

Next, we extracted and quantified cellular polyamines from the wild type and the Δ*vibF* mutant. Under the conditions of our experiment, *V*. *cholerae* can synthesize the polyamines putrescine, diaminopropane, cadaverine, and norspermidine. It does not synthesize spermidine, but can import it from the media which contains spermidine. There was no difference in norspermidine levels between wild type and Δ*vibF*, indicating increased levels of cellular norspermidine is not the cause of increased biofilms in the Δ*vibF* mutant (S2 Fig). It remained possible that cellular norspermidine levels did indeed increase initially; however, excess norspermidine was exported out of the cell to maintain homeostasis. To determine whether the increase in biofilm formation seen in the Δ*vibF* mutant is due to extracellular norspermidine, we quantified the polyamines in the media (S3 Fig). We did not detect any norspermidine in the spent media of either of the strains. This result is consistent with our previous report that showed *V*. *cholerae* does not secrete norspermidine [34]. In addition, other extracellular polyamine levels were similar in both of these strains; therefore, differences in levels of other extracellular polyamines cannot explain the difference in the biofilm phenotypes. Thus, the increased capacity of the Δ*vibF* mutant to form biofilms requires the presence of the *nspC* gene, but is not a result of increased norspermidine levels in the cell or the presence of norspermidine in the media.

### The NspS/MbaA signaling pathway is epistatic over NspC in regulating biofilm formation

**Fig 3:**
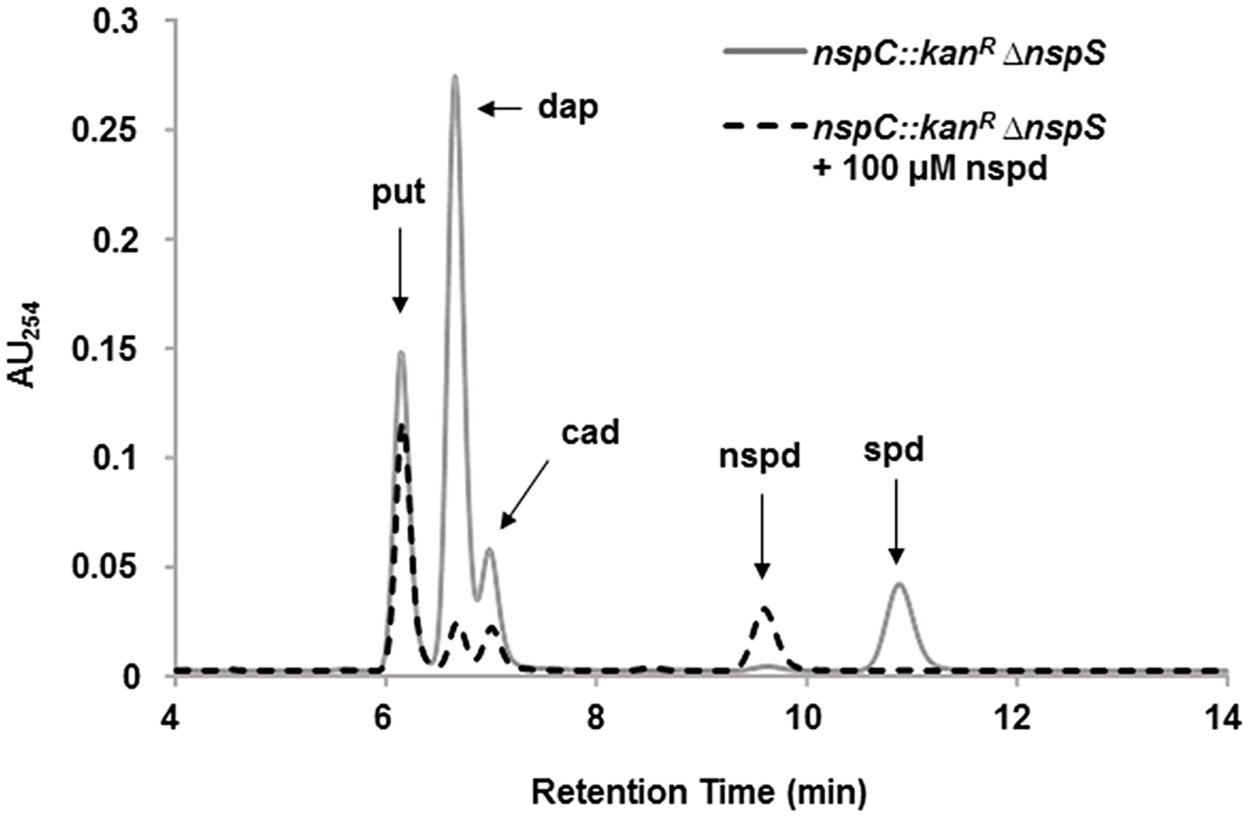
Role of NspS and NspC on cellular polyamine content in *V*. *cholerae*. Polyamine composition of *nspC*::*kan*^*R*^*ΔnspS* cells with and without exogenous norspermidine. Polyamines were extracted from cells, derivatized by benzoylation and analyzed by HPLC as described in Materials and Methods. Labeled peaks on the chromatogram correspond to putrescine (put), diaminopropane (dap), cadaverine (cad), norspermidine (nspd), and spermidine (spd). AU_254_, absorbance units at 254 nm. Only 4–14 minutes of a 40-minute run are plotted for clarity.

Previous studies have found that biofilm levels can be restored in a *nspC* mutant through the addition of norspermidine to the growth medium [10]. The authors of this study concluded that adequate norspermidine in the cell was required for normal biofilm formation in *V*. *cholerae*. Additionally, we have shown that deletion of the *nspS* gene significantly reduces biofilms; however, exogenous norspermidine is not able to restore biofilm levels in this mutant, suggesting that norspermidine synthesized and/or transported into the cell may not play a role in the ability of *V*. *cholerae* to form biofilms [8]. To ensure the drastic biofilm defect in the Δ*nspS* mutant could be complemented, we reintroduced this gene from a plasmid and compared biofilm formation to that of the wild type and Δ*nspS* mutant cells carrying the empty plasmid. Complementation with the p*nspS* plasmid restored the biofilm forming ability to the Δ*nspS* mutant (S4 Fig). To determine whether *nspC* or *nspS* is the dominant mediator of the low biofilm phenotype, we constructed a double *nspC*::*kan*^*R*^Δ*nspS* mutant. This mutant is incapable of synthesizing norspermidine; therefore, as expected the cells did not contain norspermidine (Fig 3, gray line). Putrescine, diaminopropane, and cadaverine could still be synthesized and spermidine was imported from the media. *V*. *cholerae* does not synthesize spermidine under the conditions of our experiment, but is able to import it in a process mediated by PotD1 (21). Inability to synthesize norspermidine also led to increased levels of diaminopropane, a diamine that is used to synthesize norspermidine [10]. Addition of norspermidine to the culture medium resulted in accumulation of norspermidine in the cell (Fig 3, dashed line). As we have previously shown, presence of norspermidine in the culture medium also inhibited spermidine uptake as these two polyamines are presumably imported by the same transporter, PotABCD1 [18].

**Fig 4:**
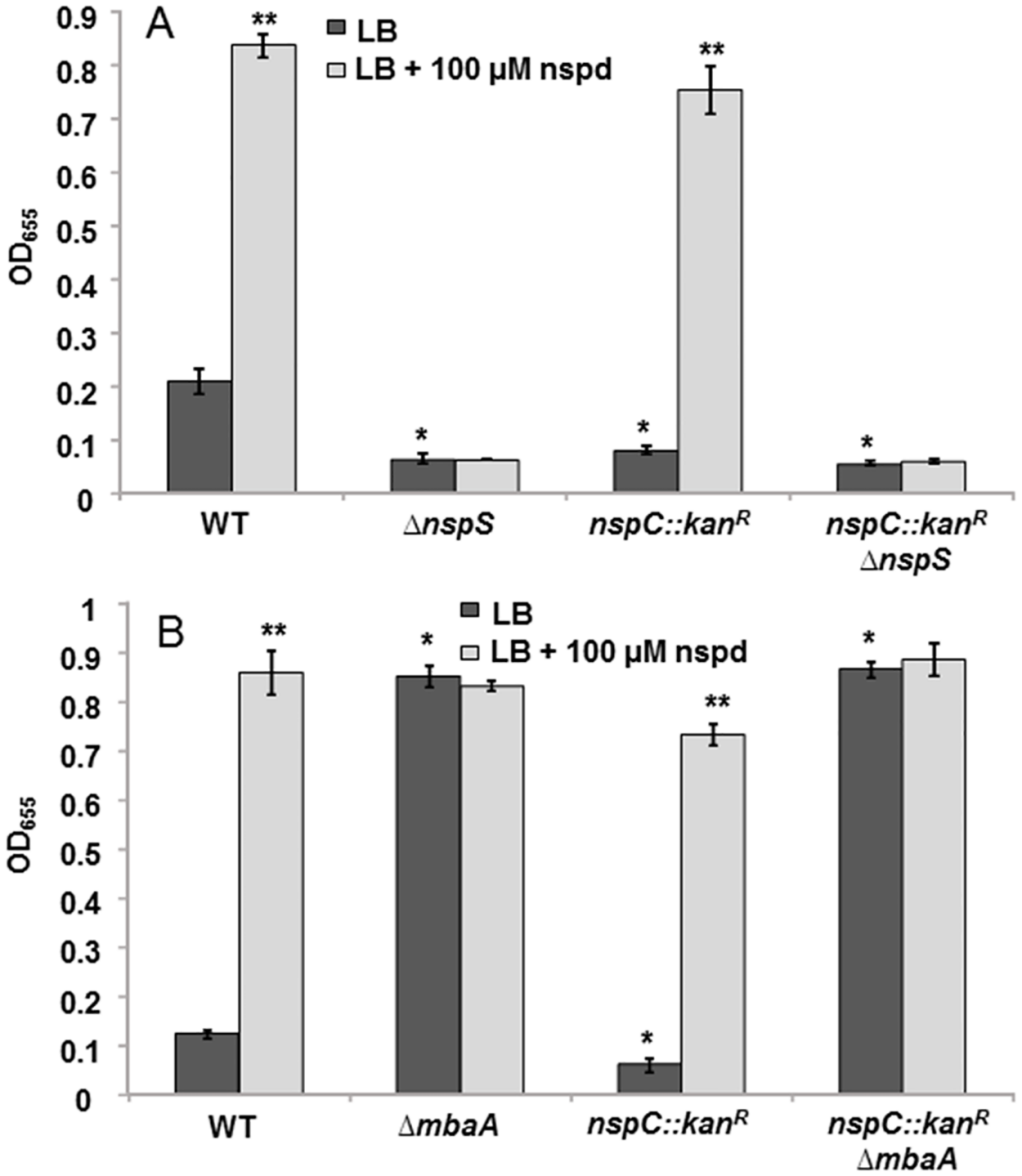
Role of NspS, MbaA, and NspC on biofilm formation in *V*. *cholerae*. **(A)** Biofilm assay of *ΔnspS*, *nspC*::*kan*^*R*^, and *nspC*::*kan*^*R*^*ΔnspS* mutations, with and without exogenous norspermidine. **(B)** Biofilm assay of *nspC*::*kan*^*R*^, *ΔmbaA*, and *nspC*::*kan*^*R*^*ΔmbaA* mutations, with and without exogenous norspermidine. Biofilms were formed in borosilicate tubes in LB broth for 18 h at 27°C and quantified as described in Materials and Methods. Error bars show standard deviations of three biological replicates. A star indicates a statistically significant difference between wild type and the mutants. A double star indicates a statistically significant difference between growth media conditions. A p-value <0.05 was considered significant. WT, wild type.

Biofilm formation by the *nspC*::*kan*^*R*^Δ*nspS* double mutant was comparable to that of the *ΔnspS* single mutant. In contrast to what was seen with the *nspC*::*kan*^*R*^ mutant, exogenous norspermidine was not able to enhance biofilm formation in *nspC*::*kan*^*R*^Δ*nspS*, despite the mutant’s ability to import norspermidine (Fig 4A). Furthermore, providing *nspS* from a multicopy plasmid to the *nspC*::*kan*^*R*^Δ*nspS* mutant was able to restore the responsiveness of this mutant to norspermidine; whereas providing *nspC* was not (S5 Fig). These results indicate that *nspS* is epistatic over *nspC* in regulating biofilm formation.

In order to corroborate these findings, we constructed an additional double mutant, *nspC*::*kan*^*R*^Δ*mbaA*. This mutant is incapable of synthesizing norspermidine and is also void of the phosphodiesterase MbaA, which is hypothesized to interact with NspS in a signal transduction pathway. MbaA is a repressor of biofilm formation and considering that reduced biofilm formation is correlated with degradation of c-di-GMP, it has been suggested that MbaA functions as a phosphodiesterase [8,37]. Indeed, we have previously shown that MbaA is able to break down c-di-GMP to pGpG, confirming its phosphodiesterase activity [18]. The *nspC*::*kan*^*R*^Δ*mbaA* double mutant formed significantly higher biofilms than wild type, with levels similar to that of the Δ*mbaA* single mutant (Fig 4B). Furthermore, complementing *mbaA* from a plasmid was able to restore the *nspC*::*kan*^*R*^ phenotype (S6 Fig). However, norspermidine was not able to increase biofilm formation in this mutant most likely because of the high levels of the MbaA enzyme expressed from the multicopy plasmid (S6 Fig). Taken together, these findings indicate that the NspS/MbaA signaling pathway is the dominant mediator of biofilm formation over synthesis of norspermidine by NspC.

We also investigated whether the increase in biofilms seen in a *vibF* mutant would still be present in the absence of NspS. Biofilm formation was significantly inhibited in a Δ*nspS*Δ*vibF* double mutant indicating that NspS is required for the high-biofilm phenotype of the *vibF* mutant (S7 Fig).

### Intracellular norspermidine is not a determinant of biofilm formation

**Fig 5:**
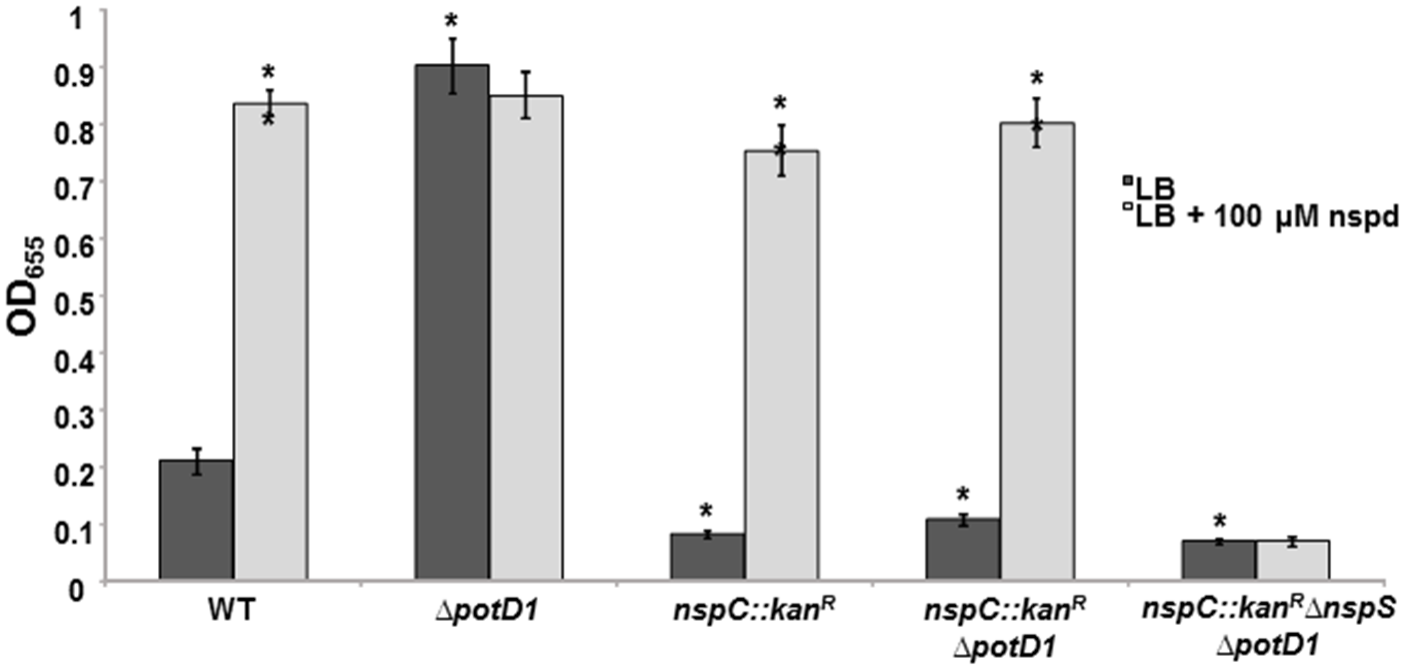
Effects of *ΔpotD1*, *nspC*::*kan*^*R*^, *nspC*::*kan*^*R*^*ΔpotD1*, and *nspC*::*kan*^*R*^*ΔnspSΔpotD1* mutations, with and without exogenous norspermidine, on biofilm formation in *V*. *cholerae*. Biofilms were formed in borosilicate tubes in LB broth for 18 h at 27°C and quantified as described in Materials and Methods. Error bars show standard deviations of three biological replicates. A star indicates a statistically significant difference between wild type and the mutants. A double star indicates a statistically significant difference between growth media conditions. A p-value <0.05 was considered significant. WT, wild type. The values for WT and *nspC*::*kan*^*R*^ in Fig 4 are the same as these experiments were performed simultaneously.

To further delineate the relative contributions of the norspermidine synthesis, transport, and signaling pathways to regulating biofilm formation, we utilized a double mutant incapable of norspermidine synthesis and transport, *nspC*::*kan*^*R*^Δ*potD1*. We have previously shown that this mutant is incapable of synthesizing or taking up norspermidine (S8 Fig); [18]. The Δ*potD1* single mutant forms robust biofilms (Fig 5 and [21]). The mechanism behind this effect is not well understood; however, we have hypothesized that it may be due to the lack of spermidine in the cell. We were able to complement the phenotype of the Δ*potD1* mutant by introducing *potD1 in trans* on a plasmid (S9 Fig). The *nspC*::*kan*^*R*^Δ*potD1* double mutant exhibited low biofilm levels, similar to the *nspC*::*kan*^*R*^ mutant rather than the Δ*potD1* mutant. Norspermidine addition did not further enhance biofilm formation by the Δ*potD1* mutant, most likely because this mutant already has maximal amount of biofilm that can be supported by these media conditions. However, both the *nspC*::*kan*^*R*^Δ*potD1* and the *nspC*::*kan*^*R*^ mutants responded to norspermidine in the environment in a manner comparable to wild type (Fig 5). Furthermore, we confirmed that NspS was responsible for this norspermidine-dependent increase in biofilms as a *nspC*::*kan*^*R*^Δ*nspS*Δ*potD1* triple mutant had low biofilm levels and was unresponsive to exogenous norspermidine. These results indicate that inability to make or transport norspermidine does not lessen responsiveness to this polyamine. Additionally, these data suggest that intracellular norspermidine is not a determinant of biofilm formation, but rather that norspermidine in the environment acts as a signal through the NspS/MbaA pathway to regulate biofilm levels.

### Increase in cellular c-di-GMP levels reverses the biofilm defect of Δ*nspS* mutants

**Fig 6:**
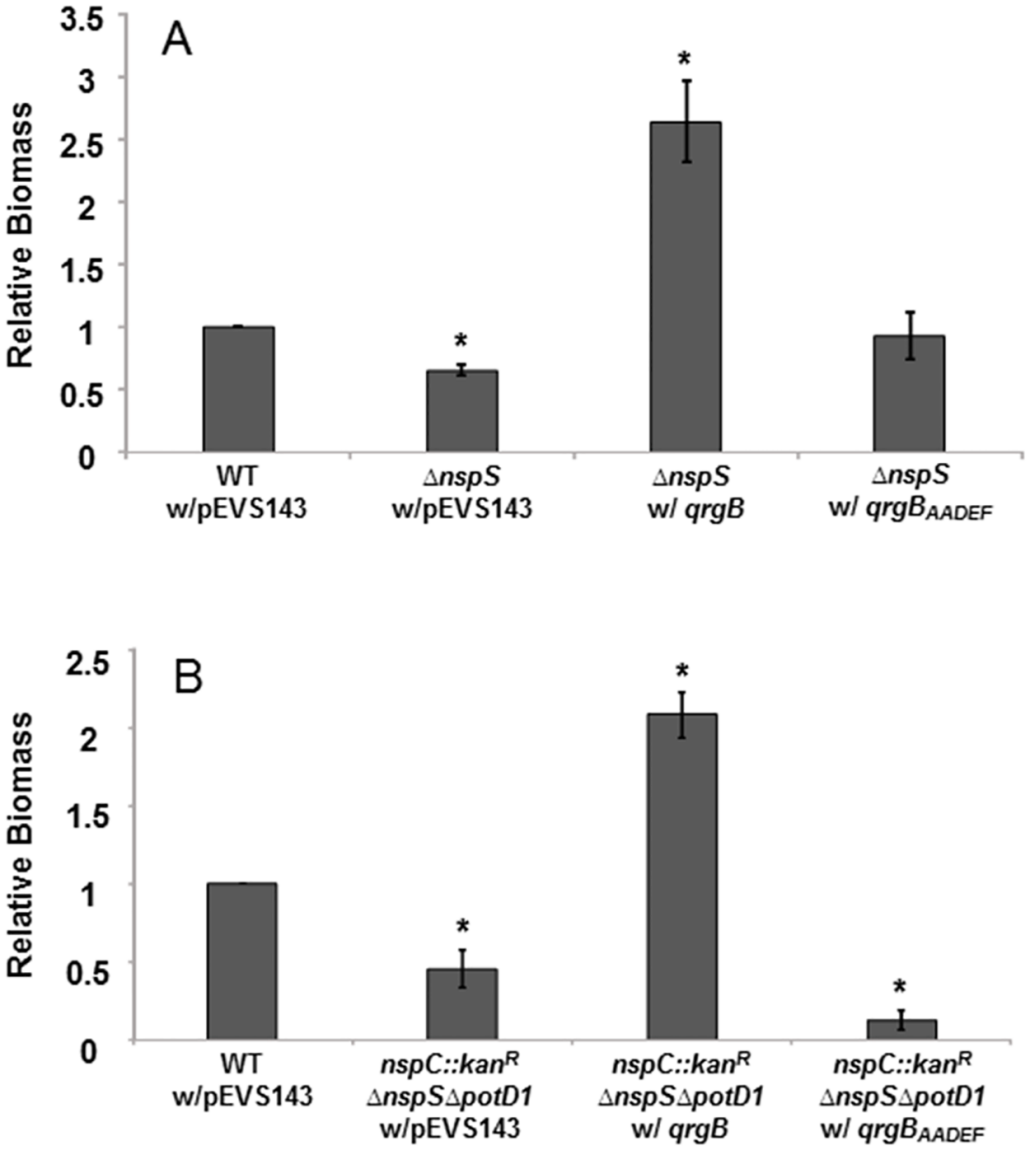
Artificially increasing c-di-GMP levels can overcome the *nspS* defect. **(A)** Biofilm assay of *ΔnspS* with *qrgB* and *ΔnspS* with *qrgB*_AADEF_ mutants. **(B)** Biofilm assay of *nspC*::*kan*^*R*^*ΔpotD1ΔnspS* with *qrgB* and *nspC*::*kan*^*R*^*ΔpotD1ΔnspS* with *qrgB*_AADEF_ mutants. Biofilms were formed in borosilicate tubes in LB broth for 18 h at 37°C and quantified as described in Materials and Methods. Relative biomass was calculated using the following equation OD_655_ mutant/OD_655_ wild type (Y-axis). Error bars show standard deviations of three biological replicates. A star indicates a statistically significant difference between wild type and the mutants. A p-value <0.05 was considered significant. WT, wild type.

NspS is hypothesized to regulate biofilm formation through its effect on c-di-GMP signaling. More specifically, NspS is thought to inhibit the EAL domain of MbaA, which is responsible for the phosphodiesterase activity that degrades c-di-GMP. Thus, inhibition of MbaA is thought to lead to increased levels of c-di-GMP, which results in increased *vps* gene expression and biofilm formation [8]. We recently quantified cellular c-di-GMP levels in the Δ*nspS* and Δ*mbaA* mutants and found that they were not different than that in the wild-type bacteria despite the drastic effects of this mutation on the biofilm phenotype. Therefore, this pathway is likely to affect local rather than global c-di-GMP pools to transmit signals [33]. We hypothesized that artificially increasing c-di-GMP levels should circumvent the negative effect of a *ΔnspS* mutation and increase biofilms. To test this hypothesis, we introduced the *qrgB* gene, encoding a diguanylate cyclase from *Vibrio harveyi*, from a plasmid into a Δ*nspS* mutant and assayed biofilm formation [28]. Overexpression of *qrgB* has previously been shown to enhance *V*. *cholerae* biofilm formation. [28]. Indeed, presence of *qrgB* in the Δ*nspS* mutant increased biofilm formation by approximately 165% (Fig 6A). To confirm that this effect on biofilm formation was due to the increase in c-di-GMP levels and not a secondary effect, we also introduced a mutated *qrgB* gene, which is incapable of synthesizing c-di-GMP, into the *ΔnspS* mutant. Indeed, the Δ*nspS* with *qrgB*_AADEF_ mutant [28], where the conserved glycines in the GGDEF motif have been replaced with alanines, exhibited biofilm levels similar to that of the Δ*nspS* mutant (Fig 6A). To further confirm the effect of a *qrgB* addition on biofilm formation, we introduced this gene into the triple mutant, *nspC*::*kan*^*R*^Δ*nspS*Δ*potD1*. This mutant cannot synthesize, transport, or detect norspermidine. The *nspC*::*kan*^*R*^Δ*nspS*Δ*potD1* with *qrgB* also had increased biofilm levels by approximately 108%. However, addition of *qrgB*_AADEF_ did not rescue biofilm formation; rather, this strain exhibited even lower biofilm levels than the *nspC*::*kan*^*R*^Δ*nspS*Δ*potD1* mutant (Fig 6B). The reason for this effect is not known.

## Discussion

Previous studies on the effects of polyamines on *V*. *cholerae* biofilms have suggested that biofilm formation is enhanced by both intracellular and extracellular norspermidine [8,18,34]. In this study, we sought to determine whether norspermidine present in the cells or the environment is the main determinant that regulates biofilm formation in this bacterium. Considering norspermidine also forms the backbone of the siderophore vibriobactin, we also investigated a potential link between vibriobactin synthesis, iron acquisition, and biofilm formation.

Because *V*. *cholerae* requires iron for cellular processes, we considered that perhaps the low biofilms formed by the *nspC*::*kan*^*R*^ mutant were due to the bacterium’s inability to synthesize the siderophore vibriobactin, which contains a norspermidine backbone. Under our conditions, however, the *ΔvibF* mutant that is incapable of synthesizing vibriobactin exhibited not a decrease but rather a significant increase in biofilm formation. The *nspC*::*kan*^*R*^Δ*vibF* double mutant formed low levels of biofilm, comparable to the *nspC*::*kan*^*R*^ single mutant. Thus, inhibition of vibriobactin synthesis increases biofilm formation; however, this increase is nullified through inhibition of norspermidine synthesis. Therefore, *nspC* is epistatic over *vibF* in regulation of biofilm formation and the inability to synthesize vibriobactin does not diminish biofilm capability under our experimental conditions. We also found that the biofilm forming ability of a *viuA* mutant, which is capable of synthesizing but not utilizing vibriobactin, was unaffected. Although iron limitation has been shown to inhibit biofilm formation in *V*. *cholerae*, a finding we confirmed in this study, the media utilized for these assays contains sufficient iron. In addition, *V*. *cholerae* has alternative iron uptake pathways that are presumably used under these conditions [19]; therefore, it is not surprising that inability to utilize the vibriobactin pathway to acquire iron did not inhibit biofilm formation. Furthermore, as both *vibF* and *viuA* mutants are unable to utilize iron through the vibriobactin pathway, they are likely to have similar levels of iron. Therefore, the difference in the biofilm phenotype between these two mutants cannot be explained by iron availability. The most likely explanation is that the cellular consequence of the inability to utilize the norspermidine to produce vibriobactin in the *vibF* mutant, but not in the *viuA* mutant, somehow leads to an increase in biofilm formation. We were unable to demonstrate an accumulation of norspermidine in the cell or in spent media that would explain this result. However, polyamine levels are tightly regulated in cells to avoid polyamine toxicity; therefore, it is also not surprising to see unchanged norspermidine levels in the *vibF* mutant [38].

Our results indicate that the NspS/MbaA signaling pathway is the main determinant of biofilm formation by *V*. *cholerae* in response to norspermidine. In the absence of the NspS periplasmic binding protein, biofilm formation is almost completely abolished and cannot be restored by norspermidine synthesis or import of exogenous norspermidine. Additionally, in the absence of the c-di-GMP phosphodiesterase MbaA, biofilm formation is very high and blocking norspermidine synthesis in this mutant does not have any negative effect on biofilm levels. Although the levels of biofilm formed by both *nspC*::*kan*^*R*^ and Δ*nspS* mutants are low, our results suggest that the presence of *nspS* is responsible for restoring biofilm formation in response to exogenous norspermidine; therefore, we conclude that intracellular levels of norspermidine are not a significant determinant of biofilm formation.

**Fig 7:**
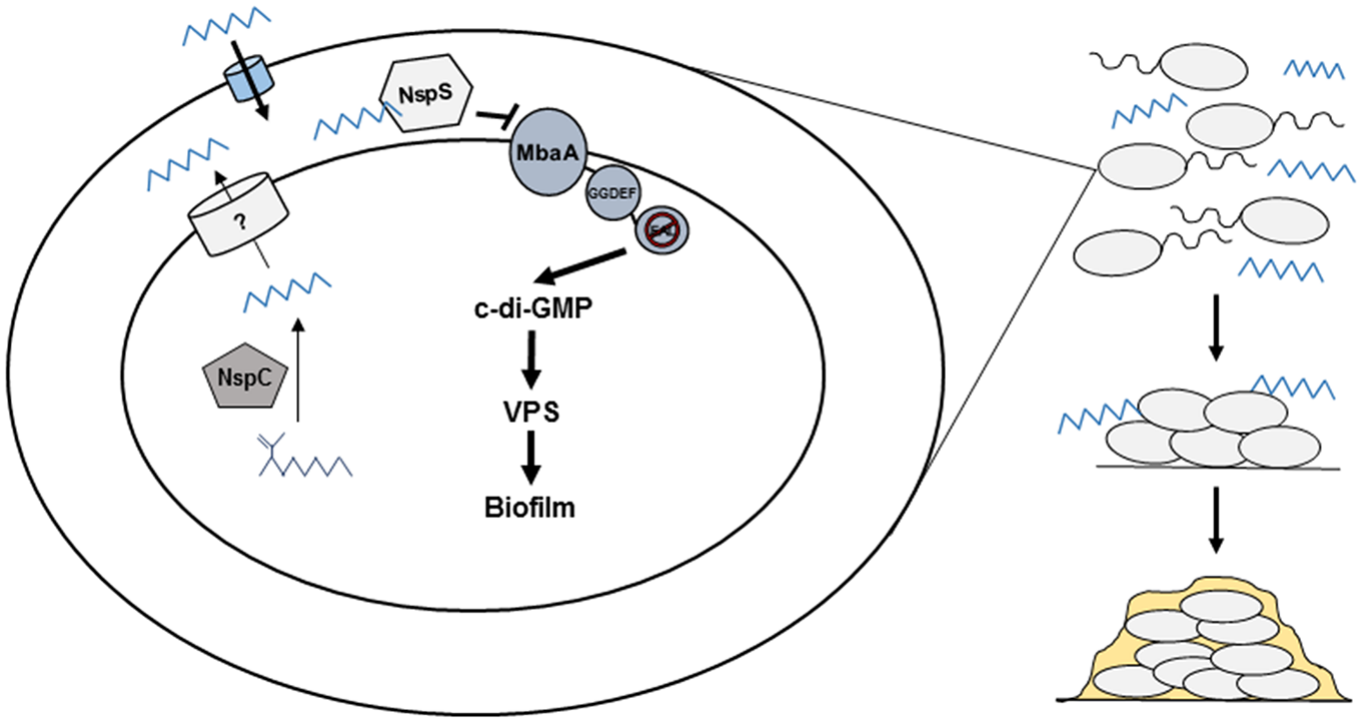
Proposed environmental model. Environmental norspermidine may primarily derive from endogenously-produced norspermidine that is released during cell lysis or exported to the periplasm by an unknown transporter. It may also be provided by nearby eukaryotic organisms. Norspermidine may also act as a quorum sensing molecule, allowing *V*. *cholerae* to detect this signal, recognize that it is in the presence of other *Vibrios*, and respond appropriately by forming the *Vibrio* polysaccharide. In this way, norspermidine may allow *V*. *cholerae* to persist in its biofilm form in its natural environment.

Although our data show that intracellular levels of norspermidine do not seem to affect biofilm levels under our conditions tested, it is clear that NspC plays a significant role in biofilm formation of *V*. *cholerae*. One explanation could be that intracellular norspermidine synthesized by NspC is exported to the periplasm, where it is then sensed by NspS to enhance biofilm formation. We have previously found that intracellular levels of norspermidine do not increase in a mutant overexpressing the *nspC* gene. Furthermore, norspermidine is not detected in the spent medium of this mutant, indicating that norspermidine neither accumulates in the cytoplasm, nor is it exported into the extracellular environment [34]. Recently, a novel ABC-type exporter, SapBCDF, was identified in *E*. *coli*, which mediates export of putrescine from the cytosol to the extracellular environment to regulate intracellular concentrations of this polyamine [39]. ABC-type transporters are located in the cell membrane; therefore, presumably, this transporter exports putrescine into the periplasm which then diffuses out of porins to the extracellular environment. *V*. *cholerae* may utilize a similar polyamine exporter to transport norspermidine from the cytosol to the periplasm in order to regulate intracellular levels. It is possible that norspermidine exported into the periplasm may either be trapped in this compartment by being sequestered by NspS, or accumulate in the periplasm because it is unable to diffuse out of the porins or both. Norspermidine in the periplasm may then act as a signal to regulate biofilm levels through NspS/MbaA (Fig 7). Therefore, periplasmic norspermidine may be a determinant of biofilm formation; however, further experiments must be conducted to test this hypothesis. This model may also explain the increased biofilms we see in the Δ*vibF* mutant, as excess norspermidine may be exported to the periplasm. The requirement for NspS for the biofilm phenotype of the *vibF* mutant is also consistent with this model.

Considering that NspS is thought to form a signaling complex with the c-di-GMP phosphodiesterase MbaA, we hypothesized that NspS mediates biofilm formation by modulating levels of c-di-GMP. Therefore, we sought to determine the relationship between NspS and c-di-GMP synthesis by providing a diguanylate cyclase *in trans* to *ΔnspS* and *nspC*::*kan*^*R*^Δ*nspS*Δ*potD1* mutants. Indeed, overexpression of the *qrgB* diguanylate cyclase corrected the low biofilm phenotype of both of these strains. Additionally, overexpressing a mutated form of the diguanylate cyclase that is unable to synthesize c-di-GMP did not rescue the low biofilm phenotype. Thus, while norspermidine acting through the NspS/MbaA signaling complex is epistatic over both norspermidine synthesis and transport, modulating c-di-GMP levels can overcome the effects of mutations in both norspermidine signaling and synthesis pathways. This is consistent with the prediction that the NspS/MbaA signal transduction system affects biofilm formation through regulating intracellular c-di-GMP levels.

Our results indicate that exogenous norspermidine is an important signal for *V*. *cholerae* to accumulate in biofilms. This suggests that norspermidine produced by prokaryotic or eukaryotic neighbors of *V*. *cholerae* signals an environment favorable for biofilm formation. Although norspermidine is a relatively rare polyamine, it has been found in a number of aquatic plants and lower eukaryotes. In aquatic plants, norspermidine is synthesized by polyamine oxidases as a byproduct of thermospermine metabolism at very low levels [13,14,16,40]. Therefore, it is unlikely that aquatic plants could produce enough norspermidine to support an association with *V*. *cholerae*. There are, however, a number of lower eukaryotes that contain higher levels of norspermidine. Norspermidine produced by some of these organisms may be released and could potentially mediate an association with *V*. *cholerae*. For example, *V*. *cholerae* has been shown to associate with *Volvox* species isolated from river water [41] and one species of *Volvox* has been reported to contain norspermidine [42]. This association presents an intriguing possibility that warrants further investigation.

Another exciting possibility is that norspermidine present in the environment of *V*. *cholerae* is primarily derived from endogenously-produced norspermidine that is released during cell lysis by members of the Vibrionales [11]. Norspermidine may then act as a quorum sensing molecule to signal that either more *V*. *cholerae* or other *Vibrio* species are present. This may be recognized as a favorable environment, leading *V*. *cholerae* to respond by forming biofilms. Norspermidine may allow *V*. *cholerae* to persist longer in its biofilm form in its natural environment, potentially in association with various eukaryotic aquatic organisms or other surfaces, as association with surfaces has been shown to improve survival of bacteria in the aquatic environments [43,44]; (Fig 7).

## Supporting information

S1 Fig Complementation of the *nspC*::*Kan*^*R*^ *mutant*. Biofilms were formed in borosilicate tubes in LB broth for 24 h at 27°C and quantified as described in Materials and Methods. Error bars show standard deviations of three biological replicates. A star indicates a statistically significant difference from wild type. A p-value <0.05 was considered significant. p*nspC*, pACYC184::*nspC*.
(TIF)

S2 Fig Polyamine composition of *V*. *cholerae ΔvibF* cells. Polyamines were extracted from cells, derivatized by benzoylation and analyzed by HPLC as described in Materials and Methods. Labeled peaks on the chromatogram correspond to putrescine (put), diaminopropane (dap), cadaverine (cad), norspermidine (nspd), and spermidine (spd). AU_254_, absorbance units at 254 nm. Only 10–28 minutes of a 40-minute run are plotted for clarity. WT, wild type.
(TIF)

S3 Fig Quantification of polyamines in spent media. (A) Polyamine composition of *V*. *cholerae ΔvibF* spent medium. (B) Polyamine composition of wild-type *V*. *cholerae* spent medium. Polyamines were extracted from media, derivatized by benzoylation and analyzed by HPLC as described in Materials and Methods. Labeled peaks on the chromatogram correspond to putrescine (put), diaminopropane (dap), cadaverine (cad), norspermidine (nspd), and spermidine (spd). AU_254_, absorbance units at 254 nm. Only 10–28 minutes of a 40-minute run are plotted for clarity. Polyamine std, polyamine standard.
(TIF)

S4 Fig Complementation of the *nspS* mutant. Biofilms were formed in borosilicate tubes in LB broth for 24 h at 27°C and quantified as described in Materials and Methods. Error bars show standard deviations of five biological replicates. A star indicates a statistically significant difference from wild type. A p-value <0.05 was considered significant. p*nspS*, pACYC184::*nspS*.
(TIF)

S5 Fig Role of NspS and NspC on biofilm formation of *V*. *cholerae*. Biofilm assay of *nspC*::*kan*^*R*^*ΔnspS* with p*nspS* or p*nspC*, with and without exogenous norspermidine. Biofilms were formed in borosilicate tubes in LB broth for 24 h at 27°C and quantified as described in Materials and Methods. Error bars show standard deviations of three biological replicates. A star indicates a statistically significant difference between growth media conditions. A p-value <0.05 was considered significant. p*nspC*, pACYC184::*nspC*; p*nspS*, pACYC184::*nspS*.
(TIF)

S6 Fig Role of MbaA and NspC on biofilm formation of *V*. *cholerae*. Biofilm assay of *nspC*::*kan*^*R*^*ΔmbaA* with p*mbaA*, with and without exogenous norspermidine. Biofilms were formed in borosilicate tubes in LB broth for 18 h at 37°C and quantified as described in Materials and Methods. Error bars show standard deviations of three biological replicates. A star indicates a statistically significant difference between wild type and the mutants. A double star indicates a statistically significant difference between growth media conditions. A p-value <0.05 was considered significant. WT, wild type; p*mbaA*, pVC0703.
(TIF)

S7 Fig Biofilm assay of *ΔvibF*, *ΔnspS*, and *ΔnspSΔvibF*. Biofilms were formed in borosilicate tubes in LB broth for 24 h at 27°C and quantified as described in Materials and Methods. Error bars show standard deviations of three biological replicates. A star indicates a statistically significant difference between wild type and the mutants. A p-value <0.05 was considered significant. WT, wild type.
(TIF)

S8 Fig Polyamine composition of *V*. *cholerae nspC*::*kan*^*R*^*ΔpotD1* cells, with and without exogenous norspermidine. Polyamines were extracted from cells, derivatized by benzoylation and analyzed by HPLC as described in Materials and Methods. Labeled peaks on the chromatogram correspond to putrescine (put), diaminopropane (dap), and cadaverine (cad). AU_254_, absorbance units at 254 nm. Only 10–28 minutes of a 40-minute run are plotted for clarity.
(TIF)

S9 Fig Complementation of the *potD1* mutant. Biofilms were formed in borosilicate tubes in LB broth for 34 h at 27°C and quantified as described in Materials and Methods. Error bars show standard deviations of three biological replicates. A star indicates a statistically significant difference from wild type. A p-value <0.05 was considered significant. p*potD1*, pACYC184::*potD1*.
(TIF)

S1 File Source data used to generate the figures and the supplemental figures in this study. (XLSX)
