# Supplementary figures and images for: Relative contributions of norspermidine synthesis and signaling pathways to the regulation of Vibrio cholerae biofilm formation
Source: PLoS One. 2017 Oct 18;12(10):e0186291. doi: 10.1371/journal.pone.0186291 (PMC5646818; doi:10.1371/journal.pone.0186291)

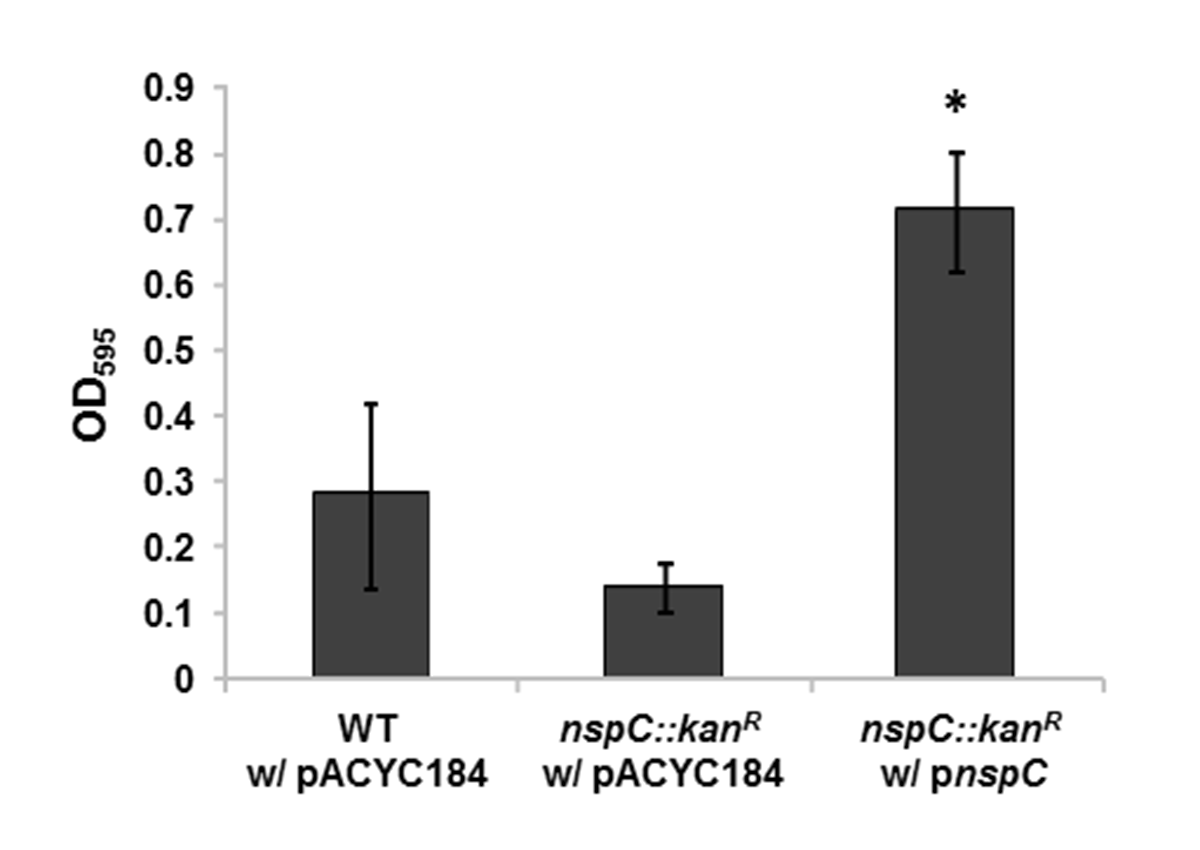

Supplement: S1 Fig — Biofilms were formed in borosilicate tubes in LB broth for 24 h at 27°C and quantified as described in Materials and Methods. Error bars show standard deviations of three biological replicates. A star indicates a statistically significant difference from wild type. A p-value <0.05 was considered significant. pnspC, pACYC184::nspC. (TIF) [file pone.0186291.s001.tif]

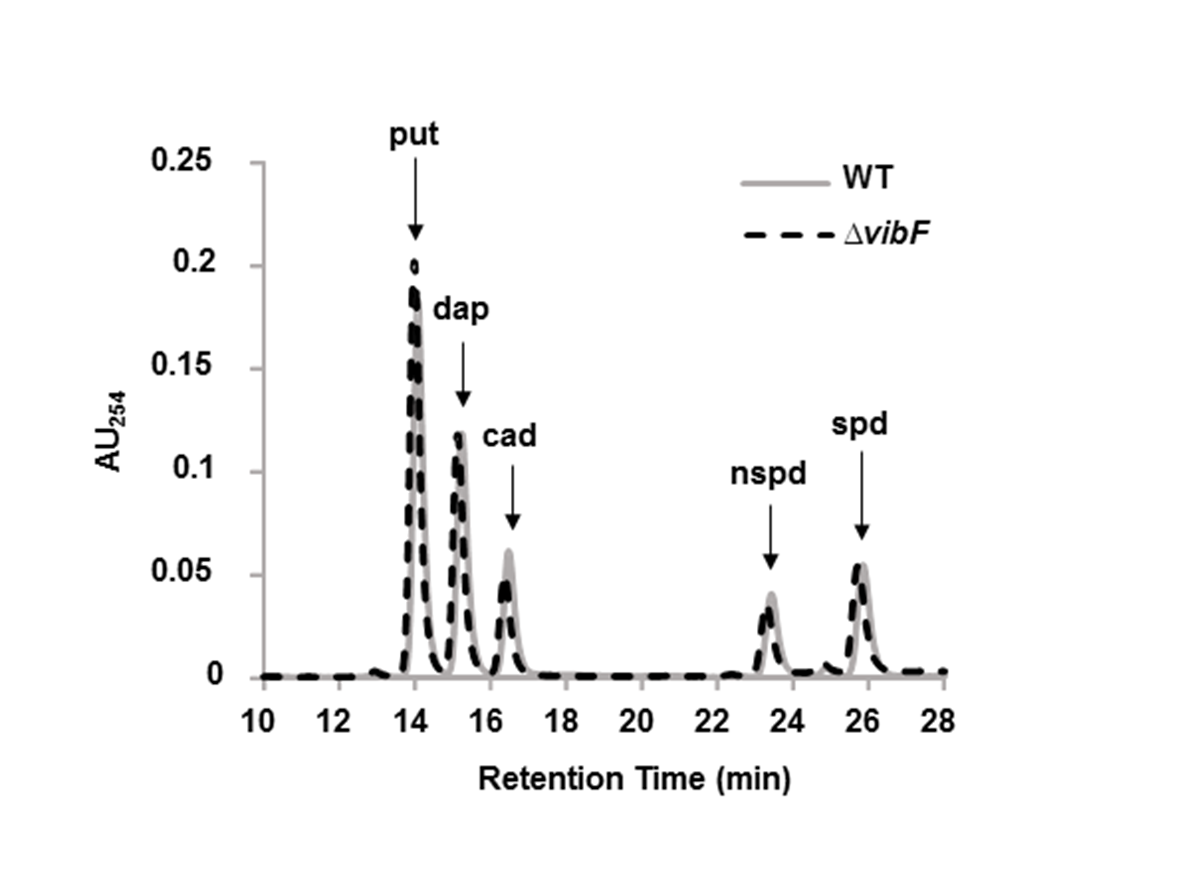

Supplement: S2 Fig — Polyamines were extracted from cells, derivatized by benzoylation and analyzed by HPLC as described in Materials and Methods. Labeled peaks on the chromatogram correspond to putrescine (put), diaminopropane (dap), cadaverine (cad), norspermidine (nspd), and spermidine (spd). AU254, absorbance units at 254 nm. Only 10–28 minutes of a 40-minute run are plotted for clarity. WT, wild type. (TIF) [file pone.0186291.s002.tif]

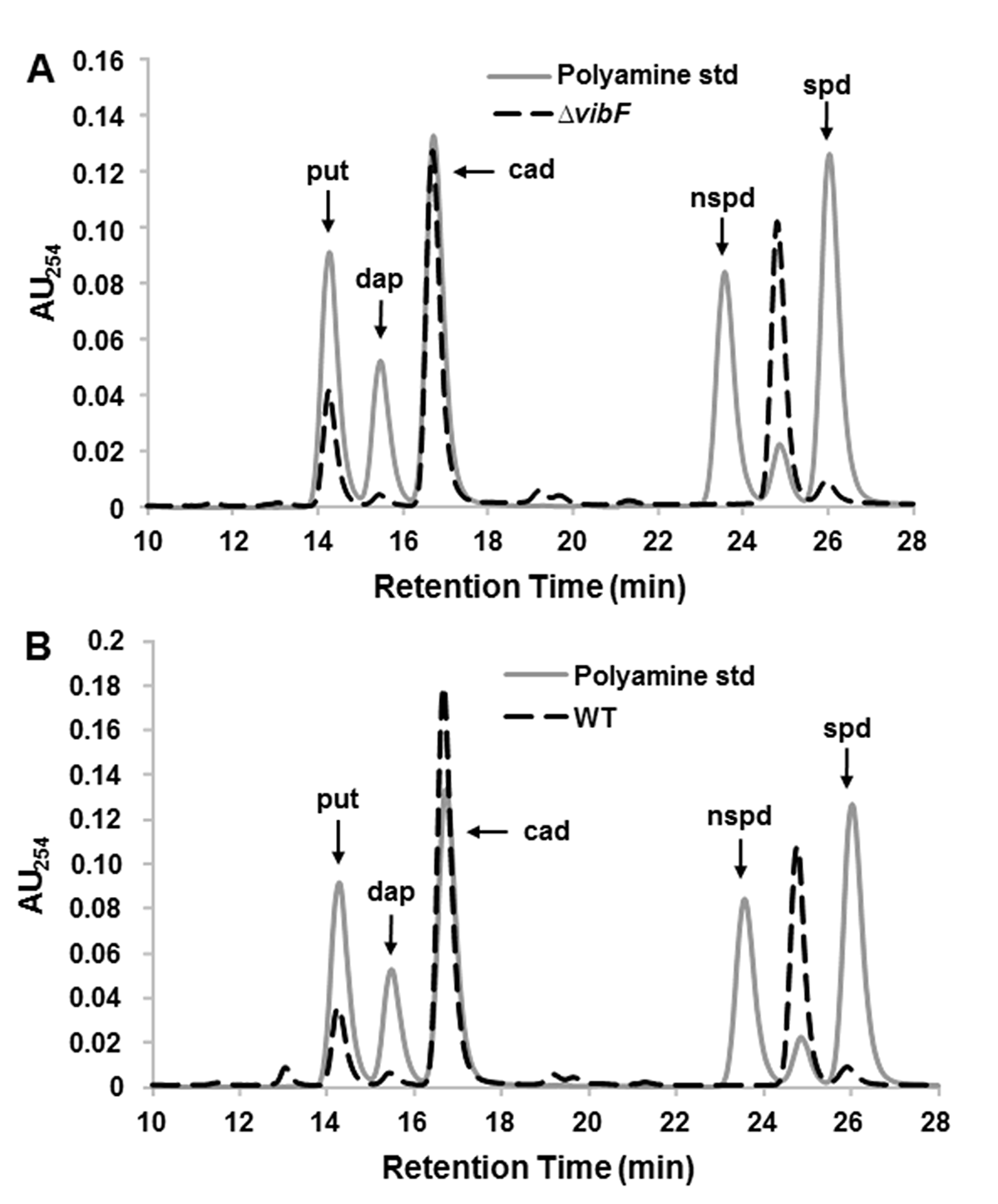

Supplement: S3 Fig — Polyamines were extracted from media, derivatized by benzoylation and analyzed by HPLC as described in Materials and Methods. Labeled peaks on the chromatogram correspond to putrescine (put), diaminopropane (dap), cadaverine (cad), norspermidine (nspd), and spermidine (spd). AU254, absorbance units at 254 nm. Only 10–28 minutes of a 40-minute run are plotted for clarity. Polyamine std, polyamine standard. (TIF) [file pone.0186291.s003.tif]

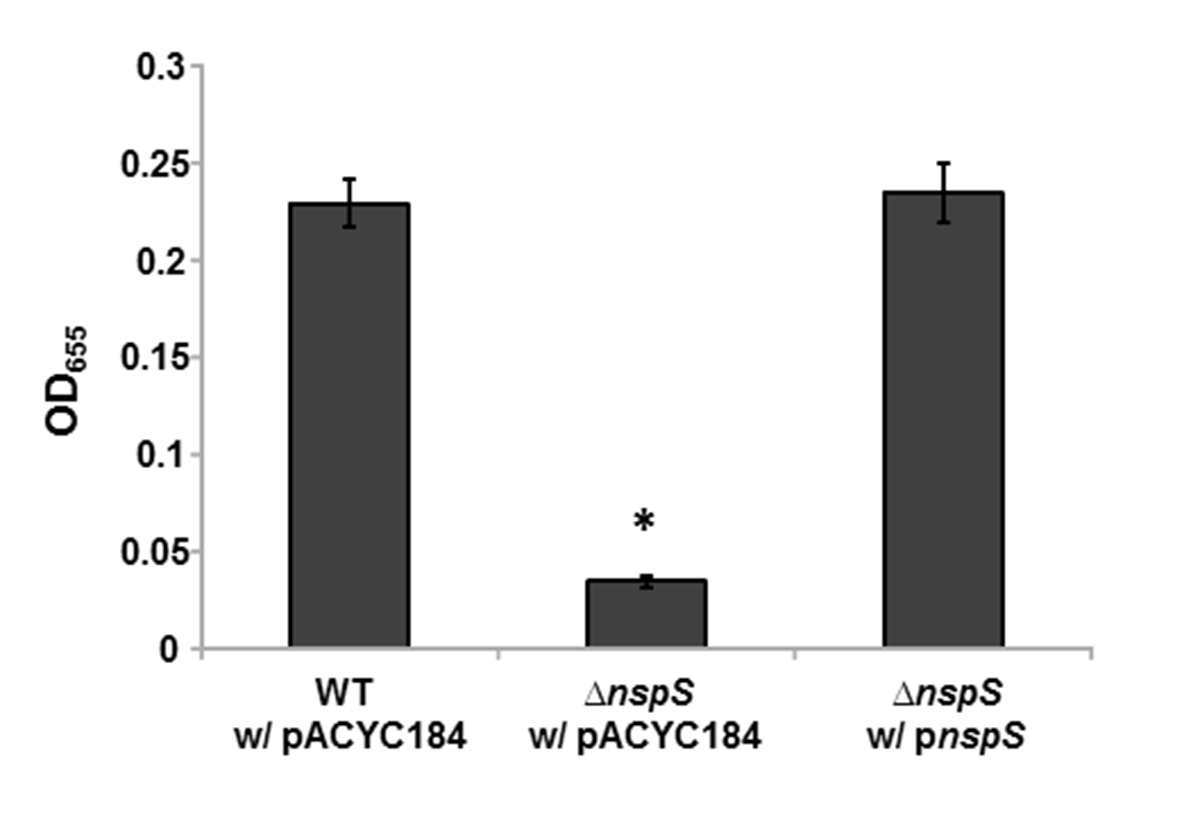

Supplement: S4 Fig — Biofilms were formed in borosilicate tubes in LB broth for 24 h at 27°C and quantified as described in Materials and Methods. Error bars show standard deviations of five biological replicates. A star indicates a statistically significant difference from wild type. A p-value <0.05 was considered significant. pnspS, pACYC184::nspS. (TIF) [file pone.0186291.s004.tif]

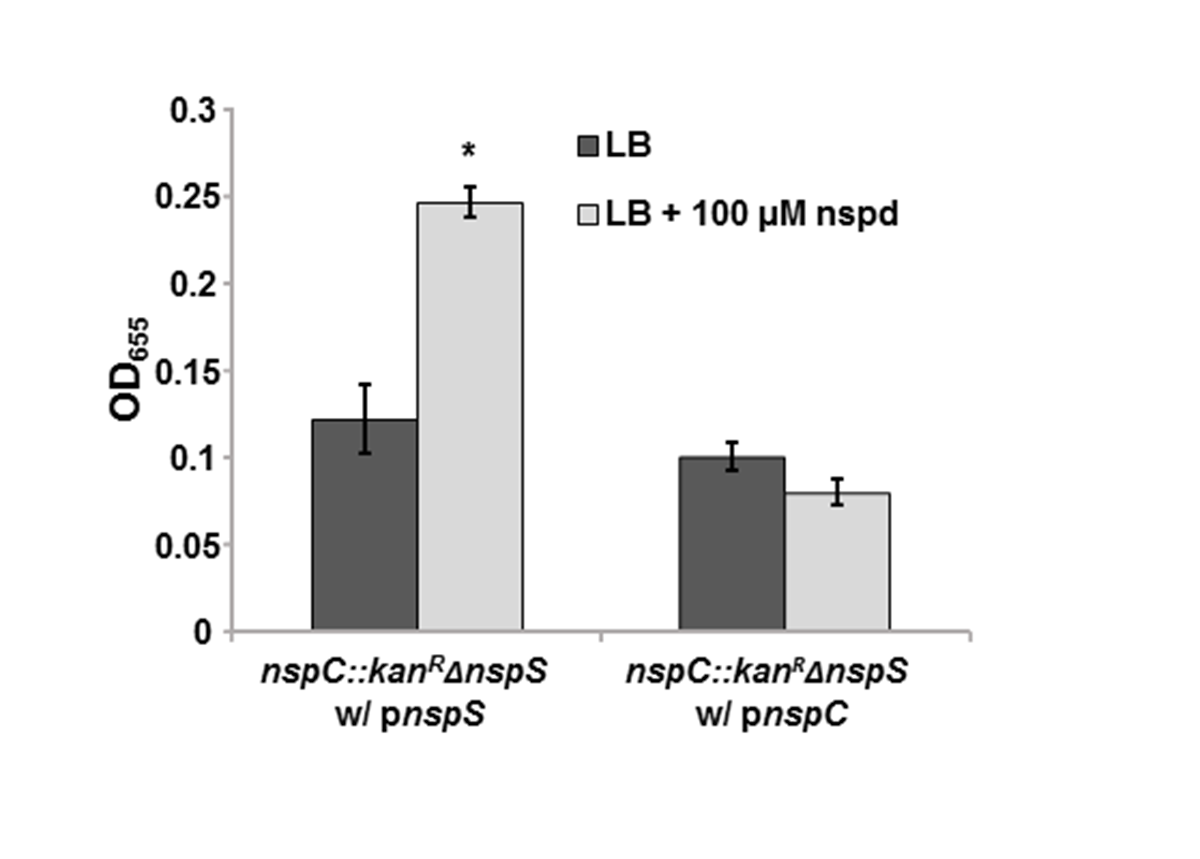

Supplement: S5 Fig — Biofilms were formed in borosilicate tubes in LB broth for 24 h at 27°C and quantified as described in Materials and Methods. Error bars show standard deviations of three biological replicates. A star indicates a statistically significant difference between growth media conditions. A p-value <0.05 was considered significant. pnspC, pACYC184::nspC; pnspS, pACYC184::nspS. (TIF) [file pone.0186291.s005.tif]

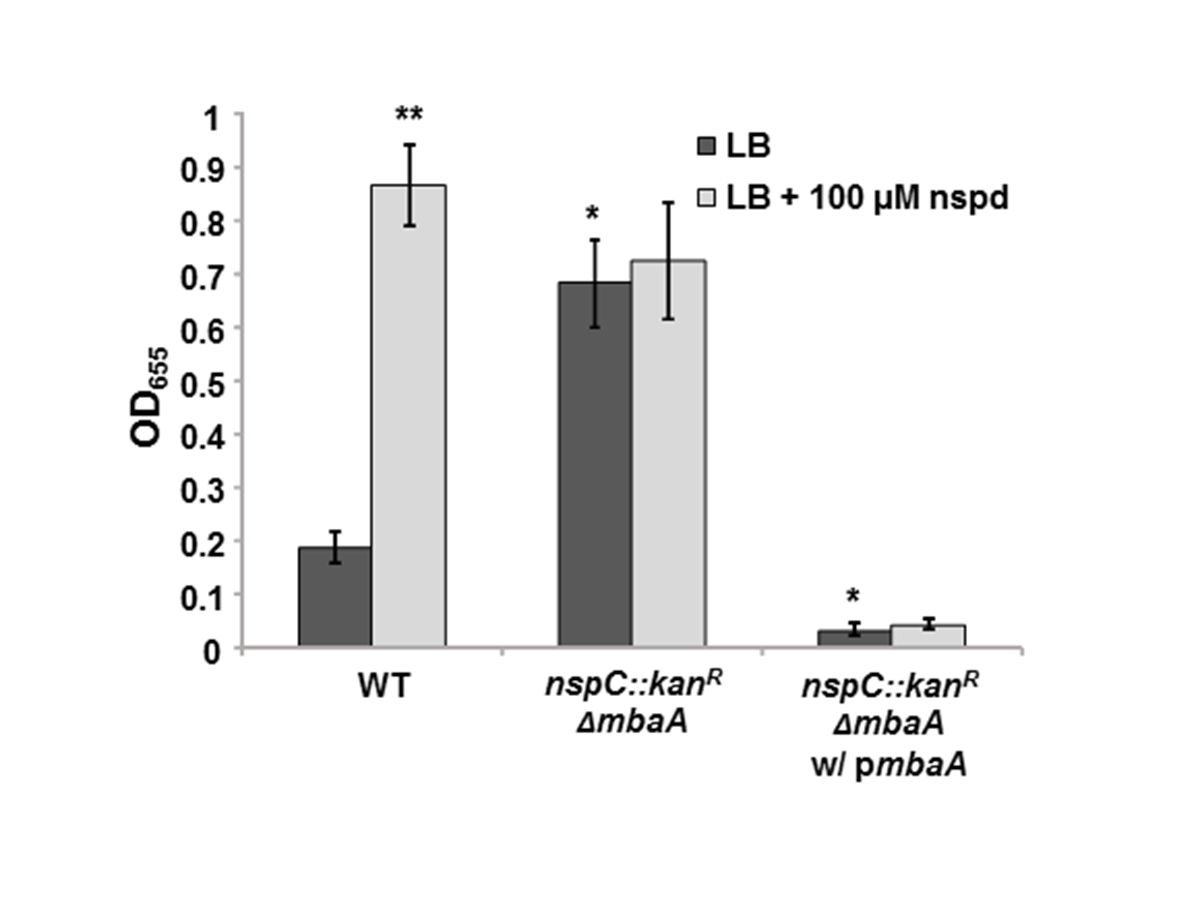

Supplement: S6 Fig — Biofilm assay of nspC::kanRΔmbaA with pmbaA, with and without exogenous norspermidine. Biofilms were formed in borosilicate tubes in LB broth for 18 h at 37°C and quantified as described in Materials and Methods. Error bars show standard deviations of three biological replicates. A star indicates a statistically significant difference between wild type and the mutants. A double star indicates a statistically significant difference between growth media conditions. A p-value <0.05 was considered significant. WT, wild type; pmbaA, pVC0703. (TIF) [file pone.0186291.s006.tif]

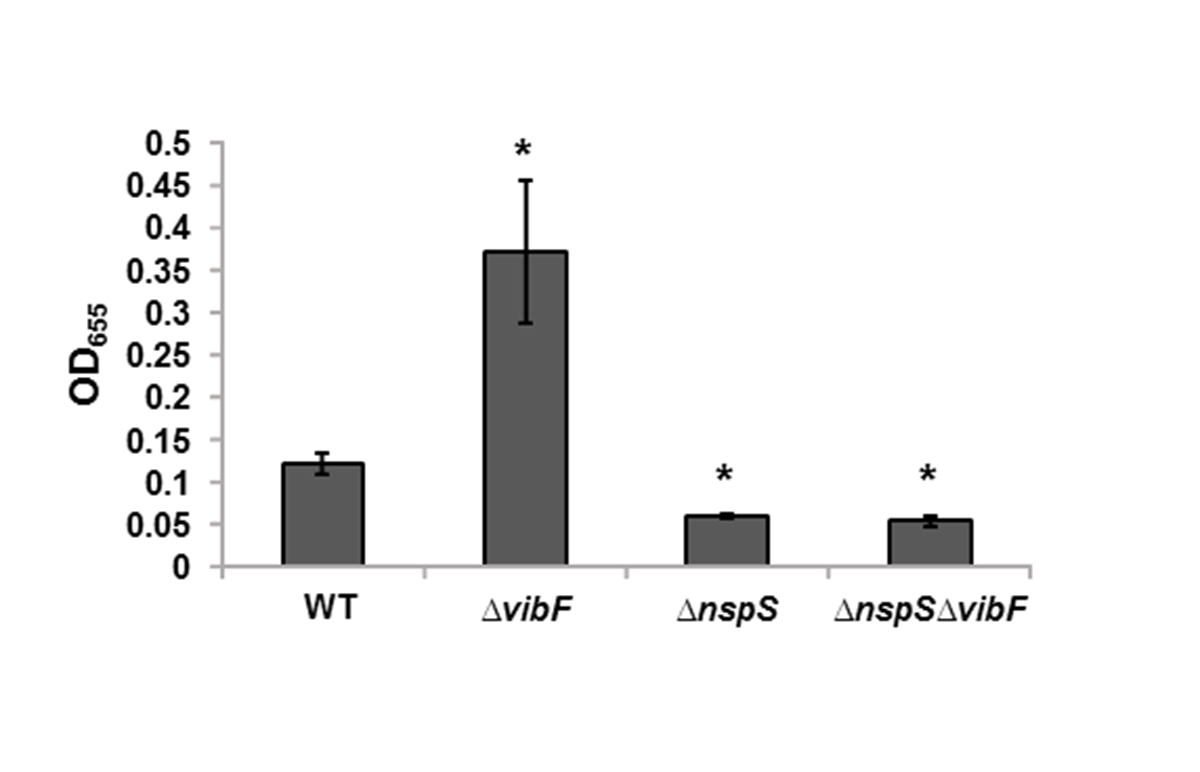

Supplement: S7 Fig — Biofilms were formed in borosilicate tubes in LB broth for 24 h at 27°C and quantified as described in Materials and Methods. Error bars show standard deviations of three biological replicates. A star indicates a statistically significant difference between wild type and the mutants. A p-value <0.05 was considered significant. WT, wild type. (TIF) [file pone.0186291.s007.tif]

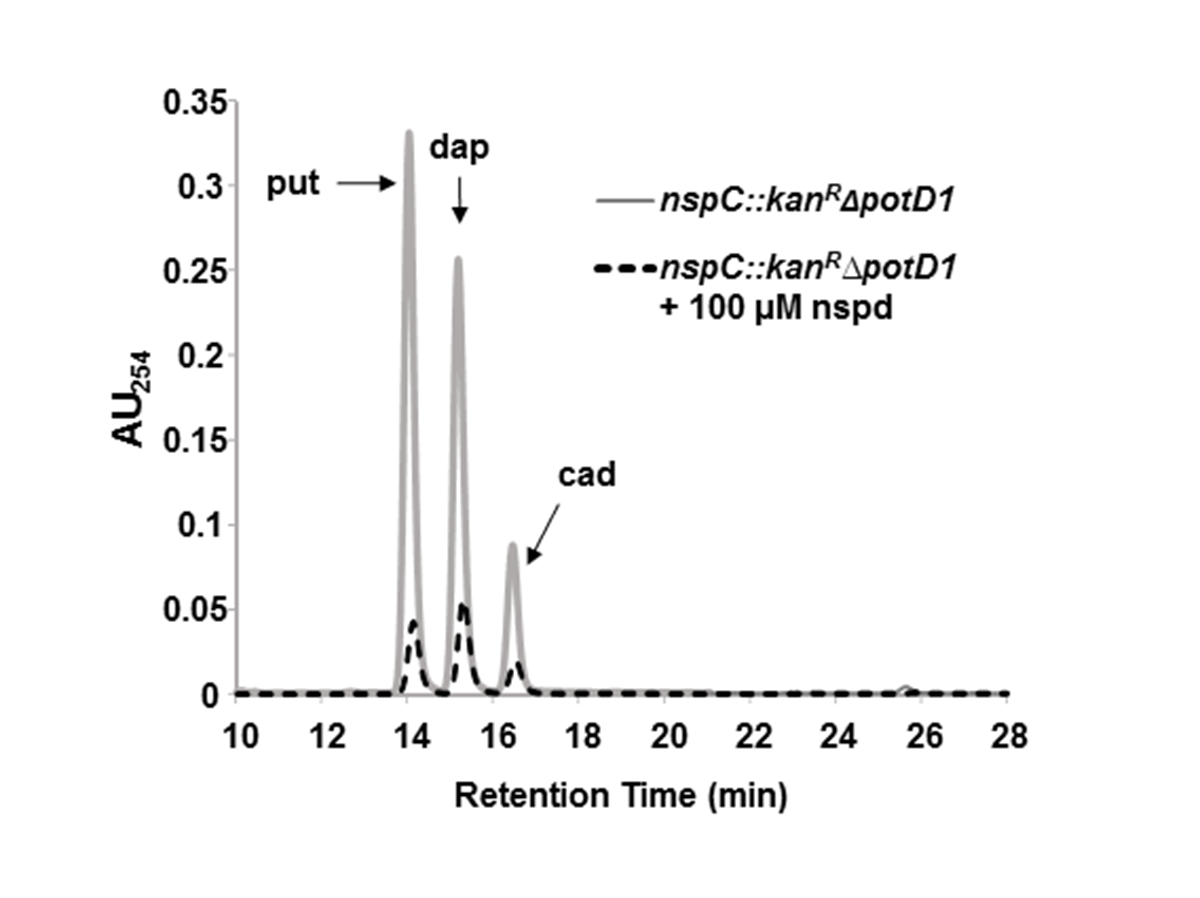

Supplement: S8 Fig — Polyamines were extracted from cells, derivatized by benzoylation and analyzed by HPLC as described in Materials and Methods. Labeled peaks on the chromatogram correspond to putrescine (put), diaminopropane (dap), and cadaverine (cad). AU254, absorbance units at 254 nm. Only 10–28 minutes of a 40-minute run are plotted for clarity. (TIF) [file pone.0186291.s008.tif]

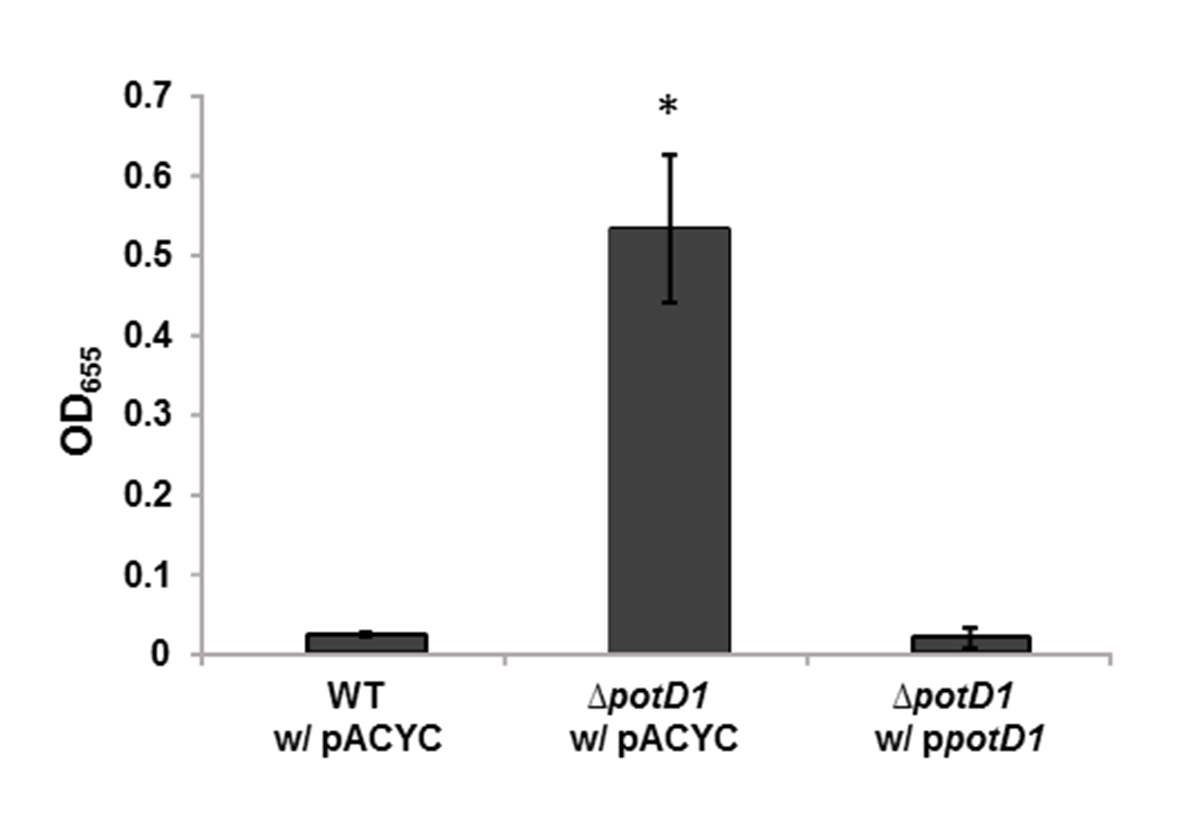

Supplement: S9 Fig — Biofilms were formed in borosilicate tubes in LB broth for 34 h at 27°C and quantified as described in Materials and Methods. Error bars show standard deviations of three biological replicates. A star indicates a statistically significant difference from wild type. A p-value <0.05 was considered significant. ppotD1, pACYC184::potD1. (TIF) [file pone.0186291.s009.tif]
